# Utility of the Health of the Nation Outcome Scales (HoNOS) in Predicting Mental Health Service Costs for Patients with Common Mental Health Problems: Historical Cohort Study

**DOI:** 10.1371/journal.pone.0167103

**Published:** 2016-11-30

**Authors:** Conal Twomey, A. Matthew Prina, David S. Baldwin, Jayati Das-Munshi, David Kingdon, Leonardo Koeser, Martin J. Prince, Robert Stewart, Alex D. Tulloch, Alarcos Cieza

**Affiliations:** 1 Faculty of Social and Human Sciences, School of Psychology, University of Southampton, Southampton, United Kingdom; 2 Health Service and Population Research Department, Institute of Psychiatry, Psychology and Neuroscience, King’s College London, London, United Kingdom; 3 Faculty of Medicine, University of Southampton, Southampton, United Kingdom; 4 Research Unit for Biopsychosocial Health, Department of Medical Informatics, Biometry and Epidemiology—IBE, Ludwig-Maximilians-University (LMU), Munich, Germany; 5 Swiss Paraplegic Research (SPF), Nottwil, Switzerland; Deakin University, AUSTRALIA

## Abstract

**Background:**

Few countries have made much progress in implementing transparent and efficient systems for the allocation of mental health care resources. In England there are ongoing efforts by the National Health Service (NHS) to develop mental health ‘payment by results’ (PbR). The system depends on the ability of patient ‘clusters’ derived from the Health of the Nation Outcome Scales (HoNOS) to predict costs. We therefore investigated the associations of individual HoNOS items and the Total HoNOS score at baseline with mental health service costs at one year follow-up.

**Methods:**

An historical cohort study using secondary care patient records from the UK financial year 2012–2013. Included were 1,343 patients with ‘common mental health problems’, represented by ICD-10 disorders between F32-48. Costs were based on patient contacts with community-based and hospital-based mental health services. The costs outcome was transformed into ‘high costs’ *vs* ‘regular costs’ in main analyses.

**Results:**

After adjustment for covariates, 11 HoNOS items were not associated with costs. The exception was ‘self-injury’ with an odds ratio of 1.41 (95% CI 1.10–2.99). Population attributable fractions (PAFs) for the contribution of HoNOS items to high costs ranged from 0.6% (physical illness) to 22.4% (self-injury). After adjustment, the Total HoNOS score was not associated with costs (OR 1.03, 95% CI 0.99–1.07). However, the PAF (33.3%) demonstrated that it might account for a modest proportion of the incidence of high costs.

**Conclusions:**

Our findings provide limited support for the utility of the self-injury item and Total HoNOS score in predicting costs. However, the absence of associations for the remaining HoNOS items indicates that current PbR clusters have minimal ability to predict costs, so potentially contributing to a misallocation of NHS resources across England. The findings may inform the development of mental health payment systems internationally, especially since the vast majority of countries have not progressed past the early stages of this development. Discrepancies between our findings with those from Australia and New Zealand point to the need for further international investigations.

## Introduction

Few countries have made much progress in implementing transparent and efficient systems for the allocation of mental health care resources.[1] In England there are ongoing efforts by the National Health Service (NHS) to develop mental health ‘payment by results’ (PbR). Healthcare providers receive funding for every patient treated, with the level of payment determined by the category (i.e. ‘mental health cluster’) to which each patient is assigned based on clinical characteristics and assessed needs.[2] There are 21 mental health clusters, organised under ‘non-psychotic’, ‘psychotic’ and ‘organic’ domains. Although mental health PbR financially rewards providers for volumes of work and thus may increase efficiency,[2] widespread criticism has contributed to repeated delays in its rollout.[3] Monitor—the NHS regulator—has highlighted that quality of care is not incentivized because provider funding is not linked to patient recovery.[4] The overall approach of using mental health clusters to determine the level of payment for each patient has been questioned given that pilot studies demonstrated their low resource homogeneity, and inferiority to an alternative statistically-derived model in reducing the variance in resource usage.[5,6] In particular, the delayed rollout can be attributed to concerns surrounding the process of clustering patients and the validity of the mental health clusters.[3]

Perhaps taking into account the lack of evidence for the utility of diagnostically-defined clusters in predicting costs, and the subsequent exclusion of mental health services from the original Medicare system of the United States,[7–11] NHS policy-makers have pursued a ‘multi-domain’ approach to clustering. Patients are assigned to mental health clusters using the Health of the Nation Outcome Scales (HoNOS)[12] and additional risk-based items. The principal assumption of the multi-domain approach is that patients who score similarly on clusters derived from the HoNOS have similar clinical needs and incur similar health service costs.[5] The HoNOS were initially proposed for clustering purposes because they “provided a suitable platform for a shared assessment tool in that [they were] already accepted and regarded as useful across the service, and nationally accepted and validated” [13](p38). As the HoNOS are used to measure clinical outcomes in the English Mental Health Minimum Dataset, the practicality of using the scales for clustering seems justifiable. However, the HoNOS were not originally designed to predict costs of care and their utility in this prediction was not considered in the decision to use them for clustering purposes.[5,13,14] As mental health PbR depends on the ability of HoNOS-derived clusters to reliably predict treatment costs,[3] this omission means that important information pertaining to the optimal allocation of resources was not taken into account.

There is little existing evidence for the utility of the HoNOS in predicting costs for patients with mental disorders. Reports on ultimately unsuccessful attempts to implement casemix classification systems in Australia[15] and New Zealand[16] provide some support for the instrument’s utility; however, the evidence is tentative because both studies only demonstrate cost associations of mental health clusters, formed of the HoNOS and other ‘casemix’ variables such as age, ethnicity, diagnosis, and focus of care. Some studies have examined the associations of HoNOS scores with health service use as a proxy for costs, but the results are mixed with both positive [17–20] and absent [21–23] associations. These limited findings neither support nor refute the utility of the HoNOS in predicting health service costs. There is an urgent need to address this unresolved issue, since a misallocation of resources could affect patients and clinical services across England.

We carried out an historical cohort study with a one-year follow up, using a comprehensive repository of anonymised electronic patient records, to investigate the utility of the HoNOS in predicting mental health service costs. We initially considered including participants with any psychiatric disorder but there was a large discrepancy in missing HoNOS data between patients with less severe disorders attending community-based services (~25%) and patients with more severe disorders who were using hospital-based care (>65%) so we therefore limited our analysis to patients with ‘common mental health problems’, represented by the recording of an ICD-10 disorder related to (unipolar) depression, anxiety, stress, adjustment or somatic problems (F32-F48). Although we use the term ‘common mental health problems’, our sample of secondary mental health care patients differs from those patients with milder and simpler difficulties treated predominately in primary care in the UK. HoNOS items are used for clustering within mental health PbR but it is commonplace for studies to deploy the total HoNOS score in analyses. To provide a comprehensive assessment of predictive utility, we separately investigated associations of individual HoNOS items and the total HoNOS score with costs.

## Methods

### Study population

#### Data source

The data source was electronic patient records from the South London & Maudsley NHS Foundation Trust (SLaM) Biomedical Research Centre (BRC) Case Register. [24] SLaM provides comprehensive secondary mental health care to around 1.2 million residents of four relatively deprived London boroughs.[24] Via the Clinical Record Interactive Search (CRIS) system, the SLAM BRC Case Register allows secondary analysis of data from de-identified SLAM records on approximately 250,000 cases. The Case Register has received approval from the Oxford Research Ethics Committee C (reference 08/H0606/71+5) and full details of this approval process are provided elsewhere[24] Further internal approval for this project was granted by the ethics committee of the SLaM BRC.

#### Inclusion criteria

We only included patients experiencing ‘common mental health problems’, represented by the recording of an ICD-10 disorder related to (unipolar) depression, anxiety, stress, adjustment or somatic problems (F32-F48). To enhance representativeness of common mental health problems, patients also needed to have a first SLaM contact in two types of community-based services primarily geared towards the treatment of less severe problems: ‘Assessment Brief Therapy’ and ‘Mood, Anxiety and Personality Disorders’. Older adults (aged 65+) were excluded because their mental health service costs are based on a range of services not attended by working age adults (e.g. ‘Mental Health of Older Adults and Dementia’ service). The sample was followed up for one year from the start of their first treatment episode occurring within the UK financial year 2012–2013 (1 April 2012 to 31 March 2013).

### Measures

#### Sample characteristics

Sample characteristics were assessed using the following variables: age (at baseline), gender, ethnicity, marital status, area-level deprivation based on the Index of Multiple Deprivation,[25] ICD-10 diagnosis, borough where a SLaM mental health service was first accessed (not limited to the observation period), type of care at end of treatment episode, and days in contact with a SLaM mental health service in the year before baseline. The number of days between the start of the baseline treatment episode and HoNOS completion was also recorded.

#### Exposures: scores on the HoNOS at baseline

The HoNOS are a set of 12 scales measuring mental health-related problems in the domains of behaviour, symptoms, impairment, and social functioning. [12] Scores on each scale/item range from 0 (no problem) to 4 (severe problem). Although not their original purpose, the HoNOS are often operationalised as a composite 12-item scale (which provides a total HoNOS score ranging from 0–48). The composite scale is the most widely used routine outcome measure in the mental health services of the UK, New Zealand and Australia.[26] Internal consistency of the composite HoNOS is moderately high and concurrent validity with other clinician-rated instruments of symptoms and multi-domain functioning is generally good (although it is poor for self-rated instruments).[27] Several factor structures have been proposed but none of these have acceptable fit.[28] Rasch analyses demonstrate the absence of an underlying construct in the composite scale.[26,29] Analyses involving individual HoNOS items have been undertaken in many studies. [16,17,30] There is no universal agreement regarding operationalisation of the HoNOS.

#### Outcome: mental health service costs at one year follow-up

Costing data were provided through SLaM internal financial records. We chose a one year follow-up period for two reasons: (1) in PbR, the interval for review of allocation to clusters covering less severe and more common disorders is typically one year; (2) the one year period is likely to even out the seasonal effects seen in admissions and mental health problems. For each patient, the mental health service costs outcome was calculated by adding costs of treatment by any community mental health team, whether generic or specialised, and the costs of any hospital treatment, taking as the start point the start of their initial treatment episode within the observation period. Both community mental health team and inpatient costs were calculated based on unit costs calculated at the individual team/ward level using SLaM internal financial data and the total relevant activity over that financial year (total face-to-face contacts for community mental health teams and total inpatient days for wards). On account of a highly skewed distribution, the outcome was transformed into ‘high costs’ (the top 10% of costs) and ‘regular costs’ (remaining 90% of costs). This cut-off was based on research demonstrating that a minority of ‘high cost’ patients (i.e. the top 5%–10% for costs incurred) account for at least a 50% share of costs [31–33], and similar cut-off points have been used in previous studies examining mental health service costs.[34–36]. In the current data set, ‘high cost’ patients accounted for a 58% share of costs.

### Statistical analysis

#### Main analyses

All statistical analyses were undertaken using Stata 12. Descriptive statistics were used for sample characteristics. The utility of both individual HoNOS items and the Total HoNOS score (at baseline) in predicting costs (at one year follow up) were determined in separate analyses. HoNOS items were simultaneously entered into all relevant predictive models. Based on previous research showing their associations with mental health service costs,[37,38] additional adjustments were made for age, gender, marital status, ethnicity, area-level deprivation (Index of Multiple Deprivation, in quintiles, for the sample), and previous health service use (previous days in contact with a SLaM mental health service in the year before baseline). To account for possible differences in service configurations across locations, the borough where a SLaM mental health service was first accessed was an additional adjustment. To account for possible changes to HoNOS scores over time, ‘the number of days between baseline and HoNOS completion’ was also included in the models. Diagnosis was not adjusted for because patients with common mental health problems are thought to have similar needs, and preliminary analysis showed diagnosis was not related to costs.

As a precursor to the main analyses, t-tests were used to examine differences in both individual item and total HoNOS scores between ‘regular cost’ and ‘high cost’ patients. Associations of baseline HoNOS scores with ‘one-year’ costs were determined using unadjusted and adjusted odds ratios with 95% confidence intervals (CIs), modelled through logistic regression analysis. Population attributable fractions (PAFs)- representing the percentage decrease in the number of ‘high cost’ patients that would theoretically arise if a problem within a given HoNOS domain could be removed from the study population- were calculated and applied to fully-adjusted models. As the ‘aflogit’ command for PAFs is not supported for use with imputed data by Stata 12, PAFs were applied to complete-case analyses.

#### Missing data

Complete data pertaining to the costs outcome were available but HoNOS scores had sizeable missing data (i.e. 24.8%). We deployed multiple imputation by chained equations (MICE) to impute this missing data. Multiple imputation uses patterns in observed data to impute missing values, repeating this process multiple times to account for uncertainty in the imputed values.[39] MICE facilitated the sequential imputation of missing data for each HoNOS item via predictive mean matching. Imputation models included all variables known to predict missingness (i.e. means of contact, type of care at end of treatment episode, the costs outcome) and all other reported variables, apart from ‘number of days between baseline and HoNOS completion’ which also had sizeable missing data. 100 imputed datasets were created The number of imputation cycles was constrained by limited computational power; however, it resulted in the introduction of minimal standard error (i.e. Monte Carlo Error), as per guidelines.[40] Checks between imputed and original values produced no anomalies. For final analyses of imputed datasets, estimates were combined using Rubin’s rules.[41]

#### Sensitivity analyses

Two sets of sensitivity analyses were undertaken: (1) complete case analyses using the fully-adjusted logistic regression models (for comparison of results with those derived from multiple imputation); (2) fully-adjusted associations of baseline HoNOS scores with a continuous ‘one year’ costs outcome, analysed using generalised linear models (GLM) with log link functions and gamma distributions (for comparison of results with those involving the dichotomized costs outcome). The use of ‘GLM-log-gammas’ accounted for the skewed distribution of the continuous costs outcome in latter analyses.[42]

#### Supplementary analysis

Initially, we did not separate costs by admitted and non-admitted settings in analysis because such separation of costs is not prioritised in mental health PbR and only 5% of the sample had an inpatient admission in the follow-up period, limiting the potential influence of setting on the findings. However, previous international studies of casemix classification systems for mental health services have separated costs by setting. For additional comparison with these studies, we present the fully-adjusted associations of HoNOS scores with inpatient admission and community health service costs in a supplementary file (S1 Table).

## Results

### Sample characteristics

Table 1 provides a full summary of sample characteristics (*n* = 1,343). The sample was ethnically diverse with a large proportion living in deprived areas. Most participants remained under the care of CMHTs at the end of their first treatment episode within the observation period.

**Table 1:** Sample characteristics (*N* = 1343).

Variable	*n* (%)	*M* (*SD*)	*Median (25*^*th*^, *75*^*th*^ *centile)*
Age^1^		39.4 (11.9)	40.0 (29.0–49.0)
Missing	0		
Gender			
Male	586 (43.5)		
Female	757 (56.4)		
Missing	0		
Ethnicity			
White	677 (50.4)		
Black	248 (18.5)		
Other	354 (26.3)		
Missing	64 (4.8)		
Marital status			
Single	913 (68.0)		
Married or cohabiting	227 (16.9)		
Divorced, separated or widowed	160 (11.9)		
Missing	43 (3.2)		
Index of Multiple Deprivation score		34.4 (8.89)^2^	35.0 (27.9–40.7)
Missing	4 (0.3)		
ICD-10 Diagnosis			
Mood disorder (F32- F39)	864 (64.3)		
NSS disorder (F40-F48)	479 (35.7)		
Missing	0		
Borough where service first accessed			
Croydon	7 (0.5)		
Lambeth	400 (29.8)		
Lewisham	395 (29.4)		
Southwark	541 (40.3)		
Missing	0		
Under care of CMHT at end of episode	1202 (89.5)		
Missing	0		
Days in contact with SLaM in year before baseline		26.3 (66.89)	0 (0–1.0)
Missing	0		
Total HoNOS score^3^		11.2 (5.39)	10.0 (7.0–15.0)
Missing	333 (24.8)		
Days between HoNOS completion and baseline^4^		4.5 (10.35)	0 (0–2.0)
Missing	326 (24.3)		

Notes:

^1^Age range is 18–64.

^2^This score is within the top quintile for deprivation in the UK population.

^3^Total HoNOS scores ranged from 0–32.

^4^’Days between’ ranged from 0–61.

CMHT = Community Mental Health Team; NSS = neurotic, stress-related or somatoform; HoNOS = Health of the Nation Outcome Scales; IMD = Index of Multiple Deprivation.

### Associations of baseline HoNOS scores with costs at follow-up

The distribution of costs by HONOS items is presented in Table 2. ‘High cost’ patients had significantly higher HoNOS scores than ‘regular cost’ patients on items relating to self-injury (*t* = 3.72, *p* < 0.01) and on the Total HoNOS (*t* = 2.35, *p* < 0.05).

**Table 2:** Differences in HoNOS scores between ‘regular cost’ and ‘high cost’ patients ^1^^,^^2^.

Variable	*M(SD)*			*t* (regular vs high cost)
	All patients	Regular cost	High cost	
HoNOS items				
(1) Behaviour (MV = 326; 24.3%)	0.56 (0.87)	0.54 (0.85)	0.69 (0.99)	1.61
(2) Self-injury (MV = 326; 24.3%)	0.64 (0.97)	0.61 (0.93)	0.96 (1.18)	3.72
(3)Drinking/ drug use (MV = 330; 24.6%)	0.42 (0.89)	0.42 (0.90)	0.34 (0.81)	0.92
(4) Cognitive Problems (MV = 326; 24.3%)	0.35 (0.68)	0.34 (0.67)	0.43 (0.79)	1.34
(5) Physical illness (MV = 326; 24.3%)	0.83 (1.17)	0.82 (1.17)	0.92 (1.23)	0.84
(6) Hallucinations or delusions (MV = 326; 24.3%)	0.39 (0.84)	0.38 (0.83)	0.48 (0.89)	1.12
(7) Depressive symptoms (MV = 326; 24.3%)	2.15 (0.91)	2.14 (0.91)	2.26 (0.93)	1.33
(8) Other mental health problems (MV = 326; 24.3%)	2.17 (0.98)	2.16 (0.99)	2.25 (0.97)	0.84
(9) Social relationships (MV = 326; 24.3%)	1.19 (1.11)	1.18 (1.10)	1.29 (1.19)	0.98
(10) Activities of daily living (MV = 327; 24.4%)	0.82 (1.03)	0.79 (1.02)	0.97 (1.16)	1.67
(11) Living conditions (MV = 327; 24.4%)	0.58 (1.02)	0.58 (1.02)	0.62 (1.04)	0.35
(12) Occupation and activities (MV = 328; 24.4%)	1.02 (1.14)	1.01 (1.14)	1.05 (1.21)	0.37
Total HoNOS (MV = 333; 24.8%)	11.15 (5.39)	11.01 (5.34)	12.30 (5.67)	2.35*

Notes:

^1^‘High cost’ patients were those scoring in the top 10% of the sample for SLaM mental health service costs incurred. ‘Regular cost’ patients were the remainder of the sample.

^2^Based on complete cases analyses.

HoNOS = Health of the Nation Outcome Scales. MV = Missing values.

* *p* < 0.05

Table 3 displays the associations of individual HoNOS items and the Total HoNOS score with (‘regular’ vs ‘high’) costs. Only ‘self-injury’ was significantly associated with costs, with positive associations found in analyses involving adjustment for other HONOS items (odds ratio = 1.31; 95% CI 1.09–1.59) and full adjustment for all covariates (odds ratio = 1.41; 95% CI 1.15–1.72). The Total HoNOS score was significantly associated with costs in unadjusted analysis (odds ratio = 1.04; 95% CI 1.00–1.08) but not in fully adjusted analysis (odds ratio = 1.03; 95% CI 0.99–1.07). PAFs for the contribution of HoNOS items to high costs ranged from 0.6% (physical illness) to 22.4% (self-injury), with four items removed from the PAF model due to their negative associations with costs. The PAF for the Total HoNOS score was 33.3%.

**Table 3:** Associations of individual HoNOS items and the Total HoNOS score (at baseline) with ‘regular’ vs ‘high’ mental health service costs (at one-year follow-up).

Variable	Odds Ratios (95% CI)			PAFs^1^, in % (95% CI)
	Model 1 (Crude)	Model 2 (Demographic)^2^	Model 3 (Model 2 + Service)^3^	Model 3(Model 2 + Service)^3^
HoNOS items^4^				
(1) Behaviour	1.17 (0.89–1.39)	1.10 (0.87–1.38)	1.06 (0.84–1.35)	4.5 (0.0–16.5)
(2) Self-injury	1.31 (1.09–1.59)	1.34 (1.10–1.63)	1.41 (1.15–1.72)	22.4 (9.5–33.3)
(3) Drinking/ drug use	0.84 (0.66–1.07)	0.85 (0.65–1.10)	0.86 (0.66–1.12)	-
(4) Cognitive Problems	1.11 (0.84–1.48)	1.10 (0.82–1.46)	1.08 (0.88–1.45)	3.6 (0.0–13.4)
(5) Physical illness	1.01 (0.86–1.35)	1.01 (0.83–1.29)	1.01 (0.83–1.21)	0.6 (0.0–14.5)
(6) Hallucinations or delusions	1.08 (0.63–1.91)	1.08 (0.85–1.36)	1.08 (0.85–1.38)	3.6 (0.0–12.3)
(7) Depressive symptoms	1.01 (0.79–1.28)	0.99 (0.77–1.28)	0.99 (0.77–1.28)	-
(8) Other mental health problems	0.98 (0.78–1.22)	0.97 (0.77–1.22)	0.98 (0.78–1.24)	-
(9) Social relationships	1.03 (0.85–1.26)	1.02 (0.83–1.25)	1.01 (0.82–1.23)	1.0 (0.0–20.4)
(10) Activities of daily living	1.11 (0.89–1.39)	1.11 (0.89–1.40)	1.06 (0.83–1.34)	7.1 (0.0–23.3)
(11) Living conditions	0.95 (0.78–1.16)	0.98 (0.80–1.21)	0.99 (0.80–1.22)	-
(12) Occupation and activities	0.95 (0.78–1.17)	0.94 (0.77–1.16)	0.95 (0.77–1.18)	-
Total HoNOS	1.04 (1.00–1.08)	1.04 (1.00–1.08)	1.03 (0.99–1.07)	33.3 (0.2–55.5)

Notes:

^1^Due to computational necessity, population attributable fractions (PAFs) are based on complete case analyses.

^2^Adjusted for the following demographic variables: age, gender, marital status, ethnicity, and Index of Multiple Deprivation score (in quintiles).

^3^Adjusted for the following health service variables in addition to Model 1: service borough, previous days in contact with a SLaM mental health service, and days between baseline and HoNOS completion.

^4^Items were adjusted for each other in all analyses.

HoNOS = Health of the Nation Outcome Scales.

### Sensitivity analyses

Table 4 reports (fully-adjusted) sensitivity analyses pertaining to the cost associations of individual HoNOS items and the Total HoNOS score. Complete case analyses yielded similar results to the main analyses: only the HoNOS ‘self-injury’ item was significantly associated with costs (odds ratio = 1.50; 95% CI 1.22–1.85). The Total HoNOS score was not associated with costs. In examination of the continuous costs outcome, ‘GLM-log-gamma’ analyses yielded largely similar results to the main analyses. Regarding HoNOS items, only ‘self-injury’ was significantly associated with costs (e^β^ = 1.17; 95% CI 1.01–1.36). However, a significant cost association was yielded for the Total HoNOS score (e^β^ = 1.03, 95% CI 1.01–1.06).

**Table 4:** Sensitivity analyses for adjusted models^1^ predicting mental health service costs.

Variable	Odds Ratios (95% CI)	e^β^ (95% CI)
	Complete cases^2^	Total costs (continuous)
HoNOS items^3^		
(1) Behaviour	1.05 (0.82–1.35)	1.05 (0.89–1.22)
(2) Self-injury	1.50 (1.22–1.85)	1.17 (1.01–1.36)
(3) Drinking/ drug use	0.85 (0.65–1.11)	0.91 (0.77–1.06)
(4) Cognitive Problems	1.07 (0.79–1.45)	1.04 (0.84–1.28)
(5) Physical illness	1.01 (0.83–1.23)	0.94 (0.83–1.07)
(6) Hallucinations or delusions	1.06 (0.82–1.35)	1.04 (0.87–1.25)
(7) Depressive symptoms	0.95 (0.72–1.25)	1.04 (0.88–1.23)
(8) Other mental health problems	0.99 (0.78–1.26)	0.97 (0.83–1.14)
(9) Social relationships	0.97 (0.79–1.20)	1.07 (0.94–1.22)
(10) Activities of daily living	1.07 (0.84–1.36)	1.14 (0.97–1.33)
(11) Living conditions	0.99 (0.79–1.24)	1.01 (0.87–1.17)
(12) Occupation and activities	0.96 (0.77–1.19)	0.94 (0.82–1.07)
Total HoNOS	1.03 (0.99–1.07)	1.03 (1.01–1.06)

Notes:

^1^Adjusted for demographic and health service variables listed in Table 3.

^2^ Dichotomized (‘regular’ vs ‘high’) costs outcome.

^3^Items were adjusted for each other in all analyses.

HoNOS = Health of the Nation Outcome Scales. e^β^ = Ratio of means, percentage increase in mean cost per unit increase in the predictor variable.

## Discussion

### Summary of findings

Findings pertaining to the associations of baseline HoNOS items and the Total HoNOS score with costs at one year follow up are summarised in turn. After adjustment for covariates, 11 of the 12 HoNOS items were not significantly associated with (‘regular vs high’) mental health service costs for patients with common mental health problems. The exception was the ‘self-injury’ item with an odds ratio of 1.41 (95% CI 1.15–1.72). PAFs for the contribution of HoNOS items to high costs ranged from 0.6% (physical illness) to 22.4% (self-injury). After adjustment, the Total HoNOS score was not significantly associated with costs in the main analysis, although the association was significant for total costs as a continuous outcome and in supplementary analyses which split costs by setting (S1 Table). Assuming that the observed effect was not accounted for by chance, the PAF of 33.3% demonstrated that it might account for a modest proportion of the incidence of high costs.

### Limitations and strengths

As high levels of missing HoNOS data led to their exclusion, the findings are not applicable to patients with more severe mental disorders and service needs. This reflects the challenge of collecting comparable mental health service data across diverse settings and clinical populations. But the findings are applicable to the first two mental health clusters from PbR, which cover the most common mental health problems. The findings are less applicable to patients with milder and simpler ‘common mental health problems’ treated predominantly in primary care in the UK. The proportion of missing data for other clinical measures was very high (e.g. 98.5% for the CORE-OM, which measures subjective well-being, functioning and risk)[43], and this prevented the examination of potentially important predictors of costs. It also meant that it was not possible to assess whether it was the constructs rated by the HoNOS, or the format of the HoNOS, that accounted for the mostly absent cost associations. Most data pertaining to comorbidity were missing and thus its effect could not be examined, albeit that comorbidity is closely associated with mental health service costs.[37] The modest PAFs for the contribution of exposures and covariates to high costs indicates that there are other determinants that have not been considered. The costs outcome did not capture the full range of health services typically accessed by people with common mental health problems (e.g. primary care psychological services) and stronger associations may have been yielded if it had been possible to incorporate such data.

The study benefits from its use of an established case register, which provided a large clinical sample from a defined catchment area covering a population of 1.2 million people, which is demographically and socio-economically similar to other deprived areas in London [24].It is the first peer-reviewed study to directly investigate the associations of scores on the HoNOS with health service costs. Therefore, the findings may have important policy implications for the English NHS, which has assigned a key role to the HoNOS in mental health PbR. The findings may also inform the development of mental health payment systems internationally, especially since the vast majority of countries have not progressed past the early stages of this development.[1] Although the costs outcome was limited in scope, it provided an approximation of the costs used for reimbursement purposes in mental health PbR. This augments the applicability of findings to this system. Findings pertaining to dichotomised costs outcomes enabled intuitive statistical interpretations via odds ratios and PAFs. These were compared with associations for continuous costs outcomes in sensitivity analyses, increasing the validity of conclusions.

### Comparison with other studies

Two studies, reporting on the development of casemix classification systems in Australia [15] and New Zealand [16], found that mental health clusters formed of the HoNOS alongside a range of casemix variables (e.g. age, ethnicity, diagnosis) were significantly associated with costs. However, the investigation of mental health clusters (i.e. categories to which patients are assigned based on their clinical characteristics and needs) rather than the HoNOS in these studies limits the comparisons that can be made. National differences in mental health service configurations also limit comparability. These issues aside, both of these studies had clinical samples comprising over 10,000 patients with a variety of mental disorders. It could be that their additional statistical power yielded associations that were not detectable in the present study. Their investigation of a broad spectrum of mental disorders may also explain the contrasting findings: the HoNOS is more often used in ‘moderate-to-severe’ clinical populations and may have better predictive ability in such populations than our sample which covered less severe disorders. Another explanation for the contrasting findings concerns confounding. The mental health clusters in the previous studies were partly defined using diagnosis. Diagnosis has been consistently found to be associated with costs [37] and accounted for a far greater amount of the variance in length of stay than any of the HoNOS items in a case-register study involving psychiatric inpatients.[20] Therefore, the contrasting absence of cost associations for the HoNOS in the current study may be attributable to our sole inclusion of patients with common mental health problems, which negated the potential confounding effect of diagnosis on examined associations.

Comparisons with the mixed findings from previous health service use studies [17–23] are limited by wide variations in the operationalisations of health service use outcomes in these studies (e.g. number of admissions, length of stay, outpatient clinic contacts). Moreover, the relevance of these studies to our research question is limited by their inability to provide a weighted summary of resource consumption through the use of costs outcomes. Overall, the results of the present study (mostly no associations) and previous research involving the HoNOS (mixed evidence for associations) highlight the need for further investigations of the utility of the HoNOS in predicting health service costs for patients with mental disorders.

Comparing the findings with previous research not involving the HoNOS, the association of self-injury with high costs corresponds with a previous report of an association of self-injury frequency with long-term health service costs.[44] In supplementary analyses (S1 Table) self-injury predicted both inpatient admission and community costs but yielded a larger association with the former outcome than the latter. The strong utility of self-injury in the prediction of hospital based costs has also been demonstrated in a UK population-level investigation of accident and emergency (A&E) visits by people with varying mental disorders.[45] The lack of a cost association for depressive symptoms is also noteworthy, given the sample composition. It could be explained by the limited ability of one-item scales to capture depressive symptom severity,[46] or the relatively weak association of depressive symptoms with costs suggested in previous research.[47,48]

### Implications for policy and future research

The predominant absence of cost associations for HoNOS items raises concerns about the decision by policy-makers to assign a key role to these items within mental health PbR, largely based on their presumed utility in predicting costs. These concerns are compounded by the lack of robust evidence from other studies for associations of HoNOS scores with costs,[17–19,21–23] operationalisation and validity issues [28,29], and the fact that the HoNOS were designed to measure clinical outcome rather than need for care.[49]

Our findings also highlight the need for assessments of alternative approaches to developing payment systems for mental health services. Monitor—the NHS regulator—has suggested that payments should be closely linked to agreed patient outcome standards to incentivise quality of care.[4] Although the lack of utility of diagnostic related groups in predicting mental health service costs is well-documented, [7–11] it would be feasible to investigate the predictive ability of the combination of broad diagnostic categories with clinical pathways.[49] Multi-domain approaches to patient clustering that make use of a wide range of patient-related variables alongside clinical outcome measures could also be examined. This approach has produced promising results in (ultimately unsuccessful) attempts to implement casemix classification systems in Australia and New Zealand [15,16]. The recently developed Australian Mental Health Care Classification (AMHCC) system also clusters patients using multi-domain information, incorporating the HoNOS into its casemix classes (https://www.ihpa.gov.au/what-we-do/mental-health-care). Another proposal is for clinicians to judge the most appropriate care pathway option based on the detailed assessment of problems in nine domains of mental health and 12 domains of everyday living, using the MRC Needs For Care Assessment Schedule.[3,50] As associations of self-injury with high costs in this study are in line with previous research, [44,45] further investigation of the utility of self-injury information in payment systems is warranted. This is especially important as PAF analyses demonstrated that self-injury contributed substantially more to the incidence of high costs than other HoNOS items. Further research avenues could be generated through examination of international attempts to implement payment systems for mental health services (e.g., in Australia, New Zealand, Canada and the Netherlands).[1]

Future investigations of the associations of HoNOS scores with costs should address the limitations of the present study. For example, a costs outcome derived from patient contacts with the full range of health services would add generalisability. Examining a broader costs outcome would also raise the issue of whether or not mental health services should be compensated for their involvement in patient care for heavy consumers of primary care services. Greater integration of service sectors, in financing as well as commissioning, planning and delivery, would be required to facilitate this reimbursement approach. Costs information could be collected directly from participants using measures such as the Client Services Receipt Inventory,[51] although this is less feasible in large-scale research, and the lack of data linkages between case-registers represents a further challenge.[52] A more diverse sample composition (e.g. including people with severe mental disorders, and older adults) would enable a more comprehensive assessment of the potential utility of the HoNOS in predicting costs. Discrepancies between our findings with those from Australia [15] and New Zealand [16], point to the need for further international investigations. Given that the composite HoNOS is the most widely used routine outcome measure within NHS mental health services, future investigations of their utility are both feasible and necessary.

## Supporting Information

S1 Table Associations of individual HoNOS items and the Total HoNOS score with inpatient admission and ‘regular’ vs ‘high’ community mental health service costs. (DOCX)
