# Supplementary material for: Utility of the Health of the Nation Outcome Scales (HoNOS) in Predicting Mental Health Service Costs for Patients with Common Mental Health Problems: Historical Cohort Study
Source: PLoS One. 2016 Nov 30;11(11):e0167103. doi: 10.1371/journal.pone.0167103 (PMC5130232; doi:10.1371/journal.pone.0167103)
Supplement: S1 Table — (DOCX) [file pone.0167103.s001.docx]

**S1 Table.** Associations of individual HoNOS items and the Total HoNOS score with inpatient admission and ‘regular’ vs ‘high’ community mental health service costs.^1^

| **Variable** | **Odds Ratios (95% CI)** | |
| --- | --- | --- |
|  | Inpatient admission | Community costs |
|  |  |  |
| Individual scales^2^ |  |  |
| 1. Behaviour | 1.19 (0.98 – 1.64) | 1.06 (0.85 – 1.33) |
| 1. Self-injury | 1.46 (1.11 – 1.93) | 1.27 (1.04 – 1.55) |
| 1. Drinking/ drug use | 0.98 (0.71 – 1.34) | 0.80 (0.61 – 1.06) |
| 1. Cognitive Problems | 1.09 (0.72 – 1.66) | 1.07 (0.81 – 1.43) |
| 1. Physical illness | 0.95 (0.72 – 1.26) | 1.02 (0.84 – 1.22) |
| 1. Hallucinations or delusions | 1.07 (0.76 – 1.50) | 1.14 (0.91 – 1.44) |
| 1. Depressive symptoms | 0.89 (0.61 – 1.28) | 1.10 (0.85 – 1.41) |
| 1. Other mental health problems | 0.82 (0.59 – 1.15) | 1.09 (0.86 – 1.37) |
| 1. Social relationships | 1.03 (0.77 – 1.39) | 1.18 (0.96 – 1.44) |
| 1. Activities of daily living | 1.16 (0.82 – 1.64) | 1.00 (0.80 – 1.25) |
| 1. Living conditions | 1.14 (0.86 – 1.53) | 1.04 (0.86 – 1.28) |
| 1. Occupation and activities | 1.02 (0.75 – 1.40) | 0.94 (0.77 – 1.15) |
|  |  |  |
| Total HoNOS | 1.06 (1.01 – 1.11) | 1.06 (1.02 – 1.10) |
|  |  |  |

**Notes:** ^1^Adjusted for demographic and health service variables listed in Table 3. ^2^Items were adjusted for each other in all analyses. HoNOS = Health of the Nation Outcome Scales.
